# Enrolling study personnel in Ebola vaccine trials: from guidelines to practice in a non-epidemic context

**DOI:** 10.1186/s13063-019-3487-0

**Published:** 2019-07-11

**Authors:** Edouard Lhomme, Camara Modet, Augustin Augier, Sylvain Faye, Tienhan Sandrine Dabakuyo-Yonli, Claire Levy-Marchal, Eric D’Ortenzio, Yazdan Yazdanpanah, Geneviève Chêne, Abdoul Habib Beavogui, Laura Richert, the PREVAC study team

**Affiliations:** 1 Univ. Bordeaux, Inserm, Bordeaux Population Health Research Center, UMR 1219, CHU Bordeaux, CIC 1401, EUCLID/F-CRIN Clinical Trials Platform, F-33000 Bordeaux, France; 2 The Alliance for International Medical Action, Alima, B.P.15530, Dakar, Sénégal; 3 Département de Sociologie, University Cheikh Anta DIOP, B.P. 5005, Dakar, Sénégal; 4 INSERM, Pôle de Recherche Clinique, 75013 Paris, France; 5 AP-HP, Hôpital Bichat-Claude Bernard, Service de Maladies Infectieuses et Tropicales, F-75018 Paris, France; 6 REACTing, Institut Thématique Immunologie, Inflammation, Infectiologie et Microbiologie, Inserm, Paris, France; 7 Centre de Formation et de Recherche en Santé Rurale de Mafèrinyah, B.P. 2649, Conakry, République de Guinée

**Keywords:** Clinical trials, Low- and middle-income countries, Ebola vaccine, Ethics, Trial participants, Research participants, Health workers, Site staff, Employees

## Abstract

**Background:**

Enrolling participants in clinical trials can be challenging, especially with respect to prophylactic vaccine trials. The vaccination of study personnel in Ebola vaccine trials during the 2014–2016 epidemic played a crucial role in inspiring trust and facilitating volunteer enrollment. We evaluated the ethical and methodological considerations as they applied to an ongoing phase 2 randomized prophylactic Ebola vaccine trial that enrolled healthy volunteers in Guinea, Liberia, Sierra Leone, and Mali in a non-epidemic context.

**Methods:**

On the assumption that the personnel on site involved in executing the protocol, as well as community mobilizers (not involved in the on-site procedures), might also volunteer to enter the trial, we considered both ethical and methodological considerations to set clear rules that can be shared a priori with these persons. We reviewed the scientific and gray literature to identify relevant references and then conducted an analysis of the ethical and methodological considerations.

**Results:**

There are currently no regulations preventing a clinical investigator or site staff from participating in a trial. However, the enrollment of personnel raises the risk of undue influence and challenges the basic ethical principle of voluntary participation. The confidentiality of personal medical information, such as HIV test results, may also be difficult to ensure among personnel. There is a risk of disruption of trial operations due to the potential absence of the personnel for their commitment as trial participants, and there is also a potential for introducing differential behavior of on-site staff as they obtain access to accumulating information during the trial (e.g., the incidence of adverse events). Blinding could be jeopardized, given knowledge of product-specific adverse event profiles and the proximity to unblinded site staff. These aspects were considered more relevant for on-site staff than for community mobilizers, who have limited contact with site staff.

**Conclusion:**

In a non-epidemic context, ethical and methodological considerations limit the collective benefit of enrolling site staff in a vaccine trial. These considerations do not apply to community mobilizers, whose potential enrollment should be considered as long as they meet the inclusion criteria and they are not exposed to any form of coercion.

## Introduction

The 2014–2016 Ebola virus disease outbreak in Guinea, Liberia, and Sierra Leone included more than 28,000 cases and more than 11,000 deaths [1]. The scale of the outbreak was unprecedented and prompted the development of prophylactic vaccine candidates [2–4]. A number of clinical vaccine trials were conducted in the affected countries during the epidemic [4–7]. Although these studies made many advances during the epidemic, further research is required to prevent and limit the impact of further outbreaks, such as the current outbreak in the Democratic Republic of the Congo [8]. Therefore, Ebola vaccine clinical trials continue to be conducted in a non-epidemic context.

Recruiting and enrolling participants in any clinical trial can be challenging, but this is especially true in the case of prophylactic vaccine trials; participants are volunteers from the community and do not have the specific disease, but rather are at hypothetical risk thereof. The Ebola epidemic context bred a well-documented climate of fear and mistrust [9, 10]. Therefore, setting up vaccine trials during the epidemic required the development of unique, highly sensitive communication (community engagement) strategies, specific to the socio-cultural context, to engage local populations in interventional research and thus facilitate study enrollment. One of the strategies used to inspire trust and combat rumors was the enrollment of “study staff,” i.e., local healthcare professionals working at the study site, as exemplified by the PREVAIL 1 trial in Liberia [5]. Nevertheless, in a non-epidemic context where there is no indication for emergency vaccination with vaccine candidates still being under development, the enrollment of study staff raises ethical and methodological concerns.

These considerations have been examined while setting up the Partnership for Research on Ebola Vaccinations (PREVAC) trial, a randomized phase 2 double-blind vaccine trial evaluating the immunogenicity and the safety of three prophylactic vaccine strategies against Ebola (NCT02876328). In this trial, healthy adult and children (aged ≥1 year) volunteers have been enrolled in Guinea and Liberia since March 2017, and in Sierra Leone and Mali, since respectively May and July 2018; the trial is ongoing. For this prophylactic vaccine clinical trial conducted in West Africa, study personnel of the PREVAC trial comprise the clinical study site staff and the community mobilizers, both employed and paid by the trial sponsors or their delegates. The clinical study site staff comprises the medical team, nurses, pharmacists, lab technicians, administrative personnel, logistics personnel, and communications staff. The community mobilizers are involved in recruitment and follow-up of participants within the communities. In the case of PREVAC, they were recruited based on a profile of community workers and community champions. Figures of exemplarity within their communities (e.g., football/soccer coach, nurse) were encouraged to apply for these positions because of their strong community involvement.

In this article, we examined the ethical and methodological considerations of enrolling study personnel in Ebola vaccine trials in a non-epidemic context. We then defined the most appropriate course of action for the implementation of the PREVAC trial in Guinea.

## Methods

We performed a review of the scientific literature using the PubMed electronic database, and of the gray literature [11] using Google searches for documents published on the Internet (in English and French), to identify relevant references regarding the enrollment of study personnel in clinical trials. Key words used for the literature search were “enrollment”, “study personnel”, “staff”, and “clinical trials”. For the review of the scientific literature, the selection was performed on the title and abstract, and then in a second step by reading the full article. For the gray literature, as it is impossible to screen all retrieved results from Google searches, we screened the most relevant results based on the Google relevancy ranking on the first 15 pages. The search was performed on March 2017 with a publication date limit of the same date. We then examined the methodological and ethical considerations pertaining enrolling study personnel in a prophylactic Ebola vaccine trial outside of an epidemic context.

As community mobilizers are rarely present at the vaccination sites (mainly being present only to provide information for the purposes of the participant consent process) and are not involved in the vaccination process, we assumed that some of the ethical and methodological considerations would not apply to them. Therefore, we distinguished between the two types of personnel—clinical study site staff and community mobilizers—for the evaluation of ethical and methodological considerations.

We then reached a consensus on how to proceed in the context of PREVAC in Guinea in March 2017 after studying all the ethical and methodological considerations. This decision was made in a multidisciplinary fashion involving the sponsor, the investigator coordinator of the study, the clinical trial unit responsible for the trial methodology, anthropologists, and study staff in the field.

## Results

### Assessment of the ethical and methodological considerations

We found no regulations preventing a clinical investigator or site staff from participating in a trial. No relevant biomedical literature was identified in PubMed among 325 articles retrieved. Our main sources were thus reference sources pertaining to the protection of human subjects such as the Declaration of Helsinki and the Belmont Report [12, 13], International Ethical Guidelines for Biomedical Research Involving Human Subjects [14], and the Institutional Review Board Guidebook of the Office for Human Research Protections (USA), especially Chapter 6 which concerns special classes of subjects [15].

The ethical and methodological features and challenges associated with enrolling study personnel in a prophylactic Ebola vaccine trial are summarized in Table 1.

**Table 1:** Ethical and methodological considerations according to the type of study personnel to be potentially included in a prophylactic Ebola vaccine clinical trial conducted in West Africa outside of the context of an active epidemic

Principles	Considerations	Explanation	Affected study personnel	
			Clinical study site staff	Community mobilizers
Ethical principles				
Respect for persons: no research without informed consent of those involved, respect of autonomy, the requirement to protect those with diminished autonomy	Direct or indirect undue influence regarding participation in the trial	As with other participants in a clinical trial, the participation of study staff must always be voluntary	Yes	Yes
Beneficence: do not harm and maximize possible benefits and minimize possible harms	No particular consideration: the risks and benefits of the research are the same for all the participants			
Justice: respect for the principle of equality of human beings, fair treatment during investigations	Breach of confidentiality regarding medical information	Every precaution must be taken to protect the privacy of research subjects and the confidentiality of their personal information	Yes	No
Methodological principles				
Subject selection	Eligibility criteria	As with other participants, any study staff enrolled must meet the eligibility criteria and participate in the information session, and give informed consent	Yes	Yes
Selection of an adequate control group	No particular consideration			
Number of subjects: statistical assessments of sample size	No particular consideration			
Response variables: primary and secondary endpoints	No particular consideration			
Methods to minimize or assess bias: randomization, blinding, compliance	Imperfect blinding	Maintaining blinding could be difficult due to the proximity to unblinded site personnel and potential knowledge of product-specific adverse events	Yes	No
	Dropout rate differences between arms	The study staff enrolled as participants may have access to accumulating information during the trial to which other participants do not typically have access (e.g., overall adverse event rates)	Yes	No
Analysis: the study protocol should have a specified analysis plan that is appropriate for the objectives and design of the study	No particular consideration			
Other	Disruption of trial operations	During their participation in trial activities as participants, the staff are not available to perform their professional duties	Yes	Yes

Respect for persons is a fundamental principle of medical research involving human subjects. As defined in the Belmont Report [13], respect for persons incorporates that individuals should be treated as autonomous agents and requires that subjects, to the degree that they are capable, be given the opportunity to choose what shall or shall not happen to them. This opportunity is provided when adequate standards for informed consent are satisfied. Participation must always be voluntary, and the volunteer has the right to refuse to participate in a study or to withdraw consent to participate at any time. Regarding this point, our consideration concerns the risk of direct or indirect undue influence because staff may feel pressured to join the clinical trial due to the hierarchical relationship with the employer. This consideration concerns clinical study site staff as well as community mobilizers, who are both in a dependent relationship with the employer.

The second ethical consideration is related to the principle of justice (i.e., respect of the principle of equality of human beings, fair treatment during investigations), where there is a risk of a breach of confidentiality of medical information. For example, confidentiality could be difficult to ensure in the case that a colleague has to inform a participant about a positive human immunodeficiency virus (HIV) test result. This concern, however, applies only to clinical study site staff, as community mobilizers do not work directly with other study staff.

Regarding the ethical principle of beneficence (i.e., do not harm and maximize possible benefits and minimize possible harms), no particular consideration was raised for the staff, as the risks and benefits of the research are the same for all the participants.

Regarding the methodological aspects, we identified three different points to consider. The first one is a logistical risk of disruption of trial operations due to the potential absence of the personnel for their commitment to trial activities as participants. As participants, all included personnel must adhere to the same trial procedures and follow-up as the other participants. This affects both clinical study site staff and community mobilizers, as it requires their availability as participants during working hours and conflicts with their professional duties as trial staff.

One of the methods used to minimize bias in clinical trials is blinding, which allows maintaining the same follow-up procedures and measurement of endpoints in the different randomized groups. The second methodological risk we identified concerns imperfect blinding in the case of single- or double-blind clinical trials, due to the potential knowledge of product-specific adverse events and to the proximity to unblinded site personnel. Vaccines are prepared on site in the pharmacy by unblinded personnel, and complete blinding is often impossible due to differences in color or viscosity between the active vaccine and placebo, even when using opaque syringes. Despite all precautions taken to achieve blinding, site staff are often familiar with subtle differences between the tested interventions that are not apparent to the participants.

The third methodological point concerns the risk of non-differential dropout rates, as the study staff enrolled as participants may have access to accumulating information during the trial to which other participants do not typically have access. For example, staff may be aware of the overall incidence of adverse events in the trial and are more likely to discontinue their participation in the trial on that basis. If blinding is maintained, this can lead to non-differential dropout rates between arms and therefore a loss of information and power. In the case of imperfect blinding, these dropout rates can be differential. These last two concerns do not apply to community mobilizers, who have a more distant relationship with the study site, are not involved in on-site procedures, and lack access to accumulating confidential information during the trial.

Regarding selection of subjects (eligibility criteria), which is an important aspect of a clinical trial, study staff enrolled must meet eligibility criteria, go through an information session, and give informed consent, just as the other participants do.

### Definition of the appropriate course of action for implementing the PREVAC trial in Guinea

Given the ethical and methodological concerns we have described, the study team in Guinea decided not to enroll investigators or site staff as participants in the trial. As community mobilizers are prominent figures who are trusted within the community, and given that most of the methodological and ethical factors did not apply to them, we allowed their enrollment in the trial under the following conditions: First, participation had to be voluntary and not related to the terms of employment. This fundamental principle had to always be well explained to the potential participants before consent was obtained; this was mandatory. Second, like all other participants, they had to meet the eligibility criteria and attend an information session, provide consent, and enroll in the participant tracking process. Finally, all site staff were reminded about their professional commitment to secrecy, especially pertaining to the confidentiality of medical information.

In October 2018, when the inclusion of participant in the PREVAC trial in Guinea ended, a total of 10 community mobilizers had been included in the trial, representing 13% of all community mobilizers working at Guinean sites.

## Discussion

The enrollment of study staff threatens two of the fundamental ethical principles of medical research involving human subjects: voluntary participation and privacy/confidentiality. It also raises logistical and methodological concerns that could jeopardize trial operations and blinding and result in increased dropout rates.

Our literature review has highlighted a lack of specific references regarding the participation of study staff to a trial, in both the scientific literature and the regulatory documents. Nevertheless, our review of scientific literature was limited to the PubMed database. There is currently no regulatory prohibition banning a clinical investigator or site staff from participating in a trial. Indeed, it would be difficult to draw up a general rule with regards to this. The public health but also socio-cultural context must be considered when determining the course of action that should be taken during the implementation of a clinical trial, especially in low-income settings and in emergency situations [8]. Regulatory authorities should consider making general or specific recommendations according to the study context, particularly in the case of a community trial for which the staff may meet the eligibility criteria. In the absence of specific rules, this matter should be addressed by the sponsor, and the opinion of an ethics committee on this question could also be sought before beginning the trial. Several strategies can be deployed to strengthen the protection of employees and minimize bias in this context [16]: Measures that will be implemented to protect the rights of employee participants should be described before the implementation and be reviewed by an ethics committee; supervisors should not directly recruit subordinates for research participation, or an independent party could monitor the informed consent process.

Building trust in the communities in West Africa as well as in other regions of the world is an essential step for the successful implementation of any community trial, including vaccine trials. The community engagement strategies developed and successfully implemented in previous vaccine trials in the epidemic Ebola context supported the tremendous effort to rapidly conduct much-needed health research on prophylactic vaccine candidates. In the epidemic context, the enrollment of study staff probably contributed to mobilize the communities, but this alone may not justify the implementation of such an initiative. During an epidemic, vaccination can potentially prevent the spread of a disease with high incidence and lethality, and a major benefit of vaccinating study staff could exist, provided that candidate vaccines have a risk-benefit ratio that has been sufficiently evaluated and that the study is well designed. These considerations could also apply to other emergent infectious diseases with high incidence and lethality and without effective prevention measures or available therapeutic treatments.

## Conclusion

The enrollment of study personnel can play a key role in facilitating the enrollment of participants in a community trial and may provide potential personal benefits, especially in an epidemic context where the risk-benefit ratio is likely to be different from the non-epidemic one. In a non-epidemic context, methodological and ethical considerations suggest the need for increased caution. We conclude that study personnel not involved in on-site procedures and who meet the eligibility criteria without risk of coercion can be considered for enrollment as participants. Our structured analysis provides a framework for the systematic examination of the specific pros and cons during the preparation and implementation of prophylactic vaccine trials.
